# DOKing tumor progression in ccRCC

**DOI:** 10.7150/jca.104784

**Published:** 2024-10-15

**Authors:** Christos Adamopoulos, Kostas A. Papavassiliou, Athanasios G. Papavassiliou

**Affiliations:** 1 Department of Biological Chemistry, Medical School, National and Kapodistrian University of Athens, Athens, Greece.; 2 Department of Oncological Sciences, Icahn School of Medicine at Mount Sinai, New York, NY, USA.; 3 First University Department of Respiratory Medicine, 'Sotiria' Chest Hospital, Medical School, National and Kapodistrian University of Athens, Athens, Greece.

We read with great interest the recent study by Xie et al., which showed for the first time that Docking protein 1 (DOK1) has an undiscovered role in promoting the progression of clear cell renal cell carcinoma (ccRCC) via the phosphatidylinositol-3-kinase (PI3K)/protein kinase B (AKT)/glycogen synthase kinase 3 beta (GSK3β) signaling axis 1. This finding adds valuable insights to the growing body of evidence underlining the molecular intricacy of ccRCC, a particularly aggressive and challenging to treat histological subtype of renal cell carcinoma, comprising approximately 80% of all malignant tumors found within the kidney.

The identification of DOK1 as an important tumorigenic factor opens new avenues for novel therapeutic interventions. This is especially timely given the urgent need for more effective treatment options for ccRCC patients. Despite recent advances in targeted therapies, such as tyrosine kinase, mechanistic target of rapamycin (mTOR) inhibitors, and immune checkpoint inhibitors (ICIs), the development of drug resistance and tumor heterogenicity hamper these efforts, whereas metastatic disease poses a serious clinical problem 2.

The correlation of DOK1 overexpression in ccRCC tissues with poorer patient outcomes underscores its potential as an appealing biomarker for both prognosis and therapeutic targeting. The results of the study by Xie et al. indicate that DOK1 silencing can impede tumor proliferation, migration, and epithelial-mesenchymal transition (EMT), key processes implicated in tumor growth and metastasis 1. The most exciting aspect of this research is that DOK1-targeted approaches could provide a new treatment strategy for ccRCC. DOK1 functions as a scaffold protein facilitating the assembly of multiprotein signaling complexes downstream of receptor tyrosine kinases (RTKs), hence regulating several signal transduction pathways 3, 4. For instance, it has been demonstrated that DOK1 can interact with the Src homology 2 (SH2) domains of the Ras-specific GTPase-activating protein (RasGAP) p120RasGAP modulating Ras signaling 3.

The study of Xie et al. revealed that DOK1 fosters ccRCC progression through inhibition of the PI3K/AKT/GSK3β signaling cascade, known to control cell survival, proliferation, and metastasis. DOK1 knockdown in ccRCC cells reduced activation of PI3K and AKT, as well as hindered downstream signaling through GSK3β, a protein engaged in EMT regulation 1. Therefore, future therapies aiming to target DOK1 could represent an effectual therapeutic approach. One possibility could be the design of selective small-molecule inhibitors or RNA-based therapies to block DOK1 expression or function in patients displaying high DOK1 levels. In a next step, combining such DOK1-targeted approaches with the currently available therapeutic regimens, such as ICIs, could augment the therapeutic window and overcome the development of drug resistance, commonly present in advanced ccRCC. Another therapeutic opportunity, according to the results of the study by Xie et al., could come from the exploration of epigenetic targeting assessing the highly methylated DOK1 status.

However, notwithstanding the promising findings of the study there are some limitations that need to be considered before they can be translated into clinical practice. First, although the compelling evidence of DOK1's involvement in the PI3K/AKT/GSK3β signaling pathway in ccRCC, the broader network of interactions within the tumor microenvironment (TME) remains elusive. This is of cardinal importance because of the vague DOK1 role as tumor suppressor or tumor promoting in various contexts 5-10. Thus, deciphering the DOK1-interacting signaling circuitries and the underpinning regulatory mechanisms would offer a clearer picture of its role in ccRCC. Most importantly, the conclusions drawn from *in vitro* experiments needs to be validated by *in vivo* models that will evaluate the clinical applicability of the DOK1-targeted strategy. Furthermore, the reliance on publicly available datasets, such as TCGA, while useful for large-scale analysis, introduces potential biases that may not fully capture the diversity of ccRCC cases. More localized, patient-specific data would help to confirm the relevance of these findings.

In summary, the study by Xie et al. sheds light on the role of DOK1 in ccRCC and highlights its potential as a therapeutic target. Nevertheless, further research is necessary to move these findings in the clinical setting.
